# Supplementary figures and images for: STREAK: A supervised cell surface receptor abundance estimation strategy for single cell RNA-sequencing data using feature selection and thresholded gene set scoring
Source: PLoS Comput Biol. 2023 Aug 21;19(8):e1011413. doi: 10.1371/journal.pcbi.1011413 (PMC10470905; doi:10.1371/journal.pcbi.1011413)

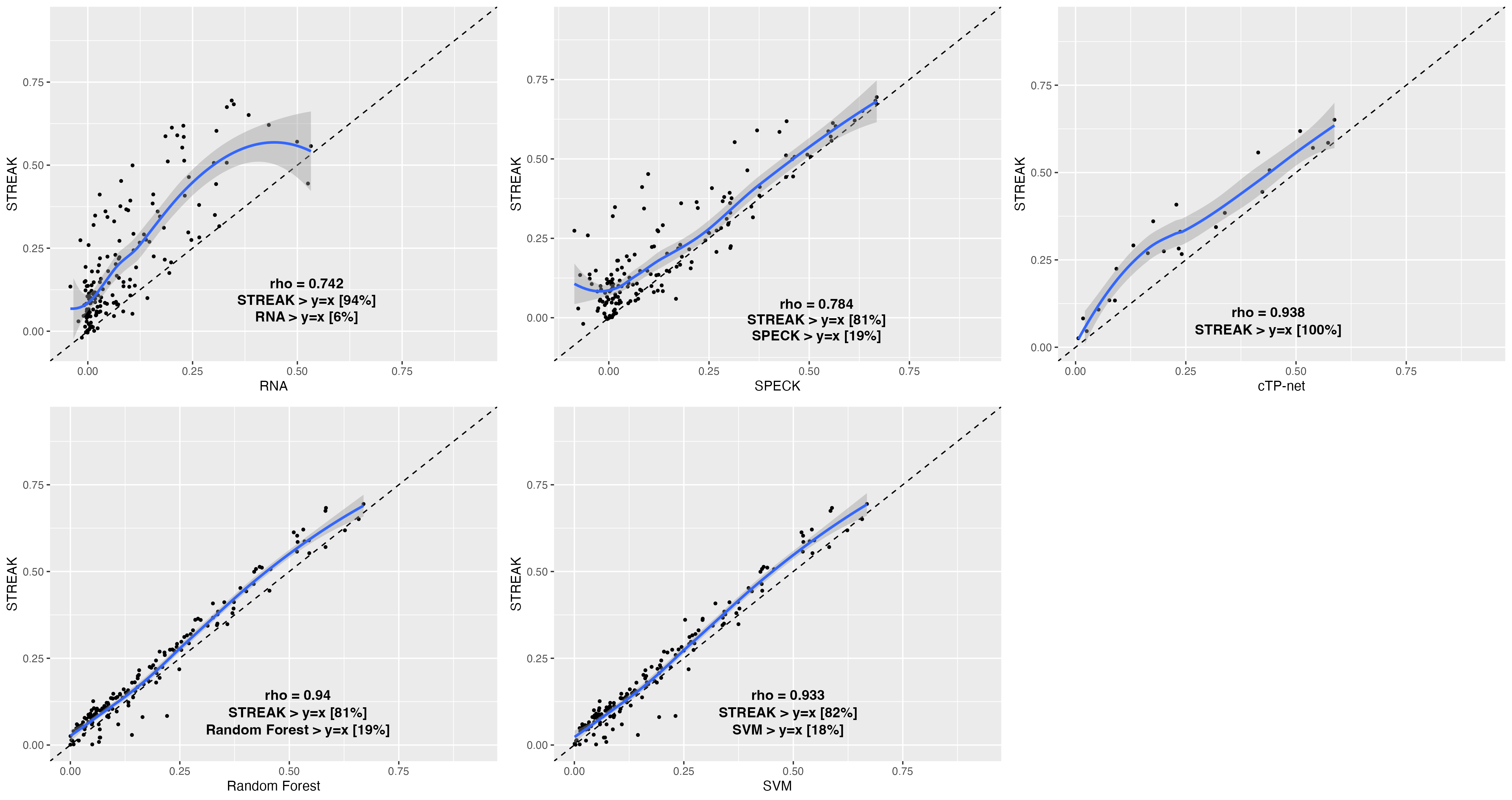

Supplement: S1 Fig — Correlation versus correlation scatter plots for the PBMC Unterman data. Each point corresponds to a receptor from a sample size of 167 receptors. (TIFF) [file pcbi.1011413.s001.tiff]

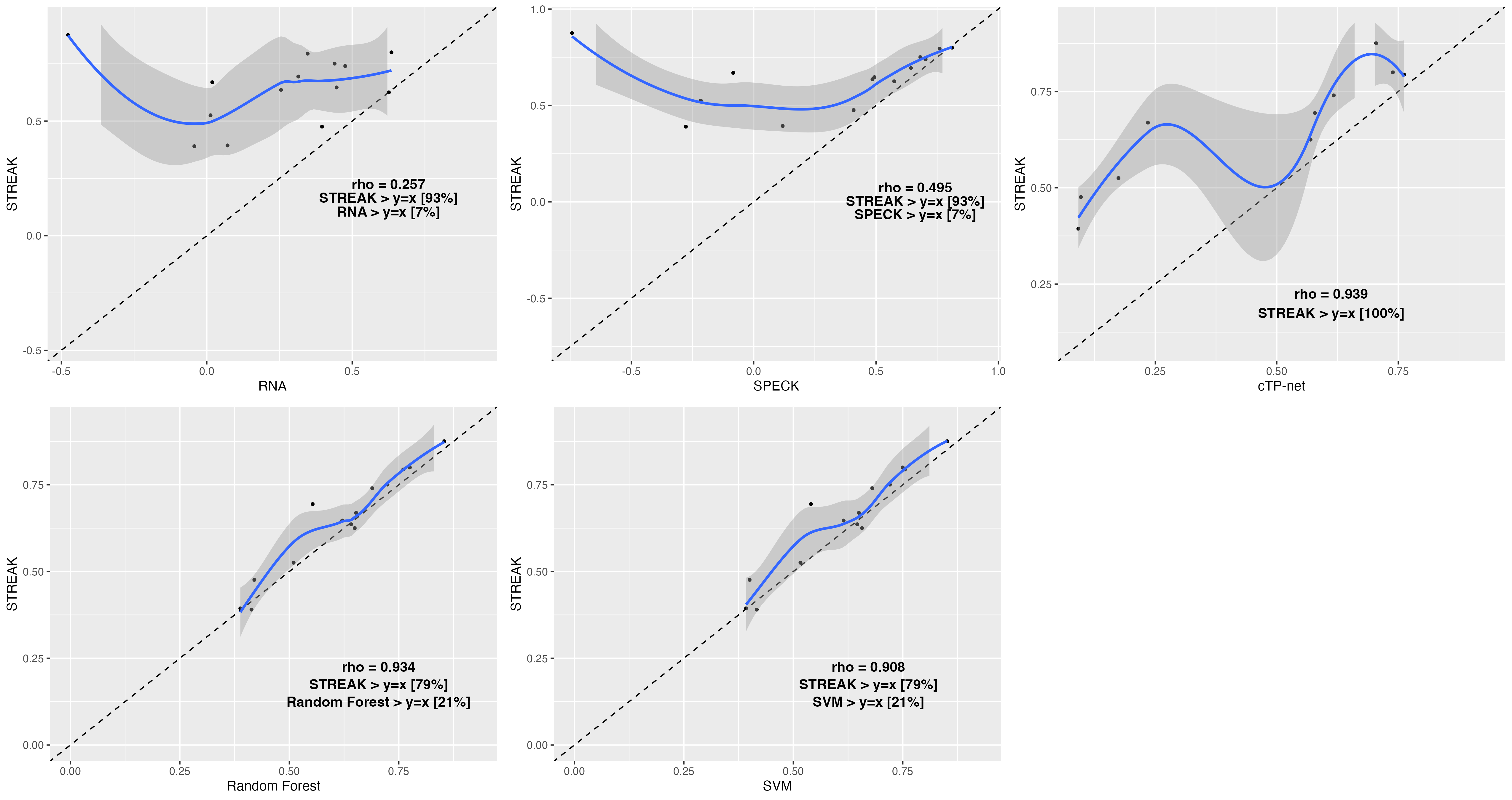

Supplement: S2 Fig — Correlation versus correlation scatter plots for the MALT data. Each point corresponds to a receptor from a sample size of 14 receptors. (TIFF) [file pcbi.1011413.s002.tiff]

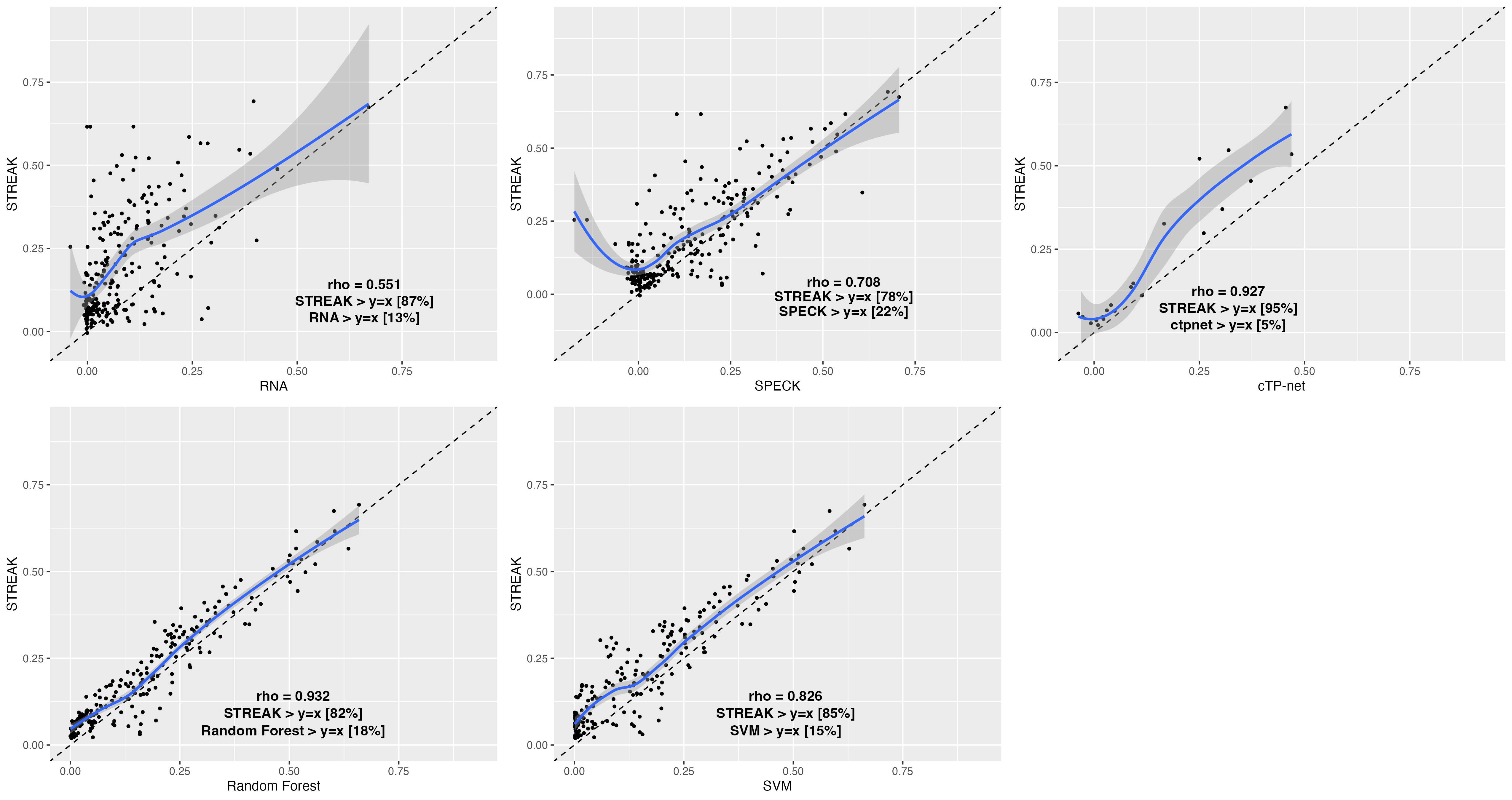

Supplement: S3 Fig — Correlation versus correlation scatter plots for the Monocytes data. Each point corresponds to a receptor from a sample size of 252 receptors. (TIFF) [file pcbi.1011413.s003.tiff]

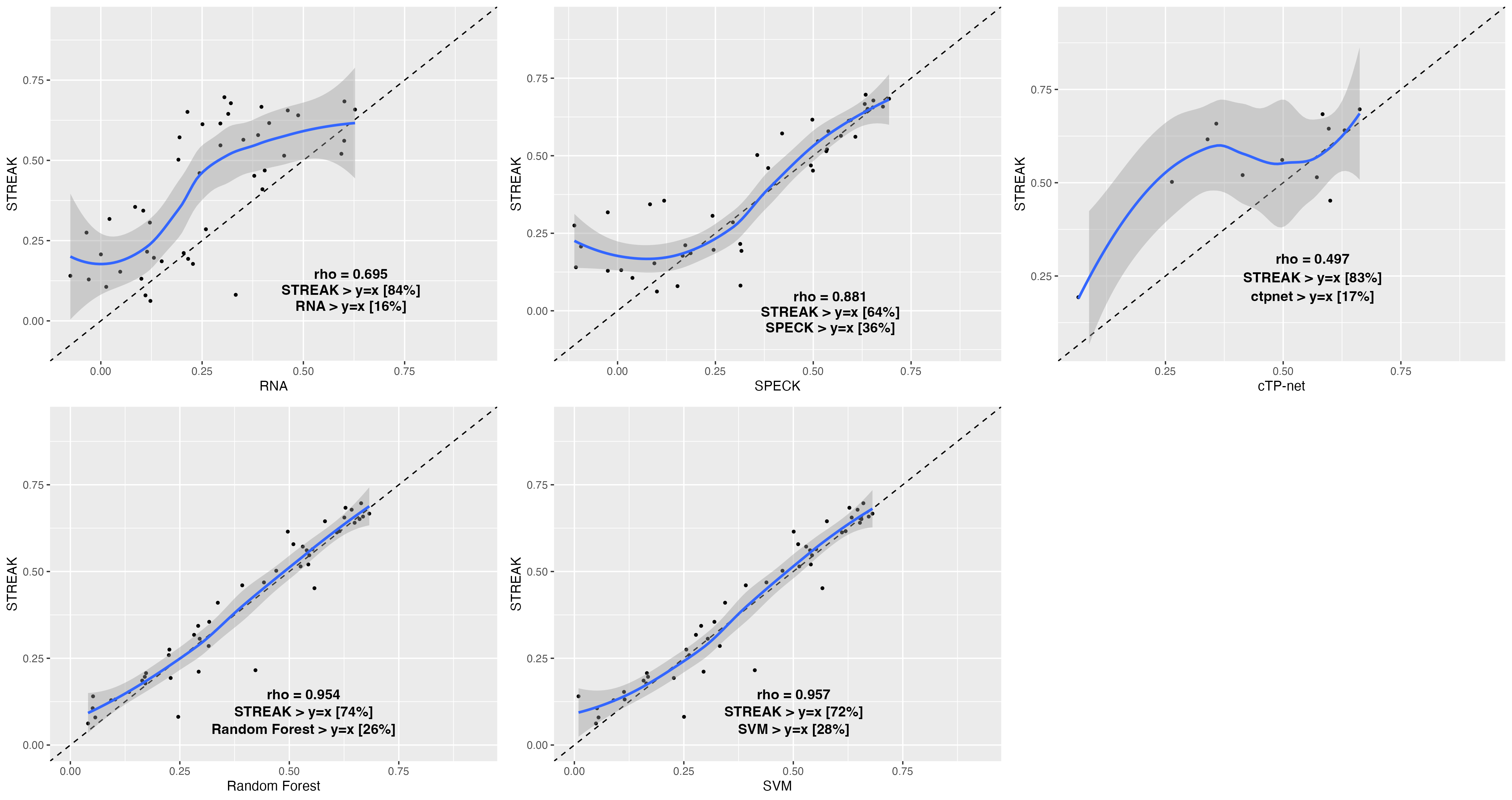

Supplement: S4 Fig — Correlation versus correlation scatter plots for the MPEM data. Each point corresponds to a receptor from a sample size of 46 receptors. (TIFF) [file pcbi.1011413.s004.tiff]

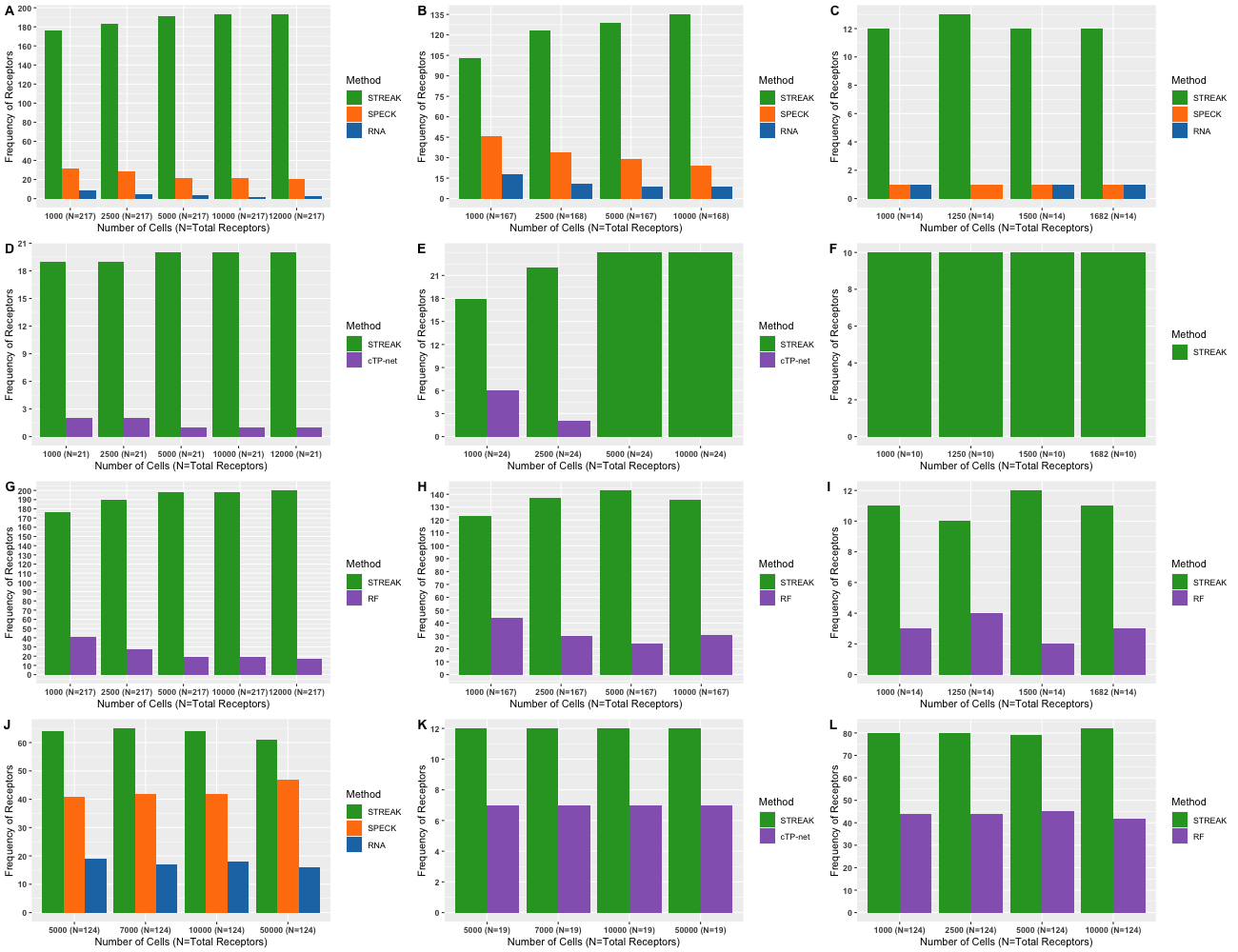

Supplement: S5 Fig — Frequency of receptors with highest average Spearman rank correlations between CITE-seq data and abundance profiles estimated using STREAK, SPECK and normalized RNA approach or cTP-net or RF for the 5-fold cross-validation approach with training data ranging from 1,000 to 12,000 cells for the Hao data (S5A, S5D, S5G), 1,000 to 10,000 cells for the Unterman data (S5B, S5E, S5H) and 1,000 to 1,682 cells for the MALT data (S5C, S5F, S5I) and 5,000 cells from the Hao data for the cross-training evaluation approach (S5J, S5K, S5L). The horizontal axis for the 5-fold cross-validation evaluation plots (S5A-S5I) indicates the number of cells used for training while the horizontal axis for the cross-training evaluation plots (S5J-S5L) indicates the number of target cells evaluated from the Unterman data. (TIFF) [file pcbi.1011413.s005.tiff]

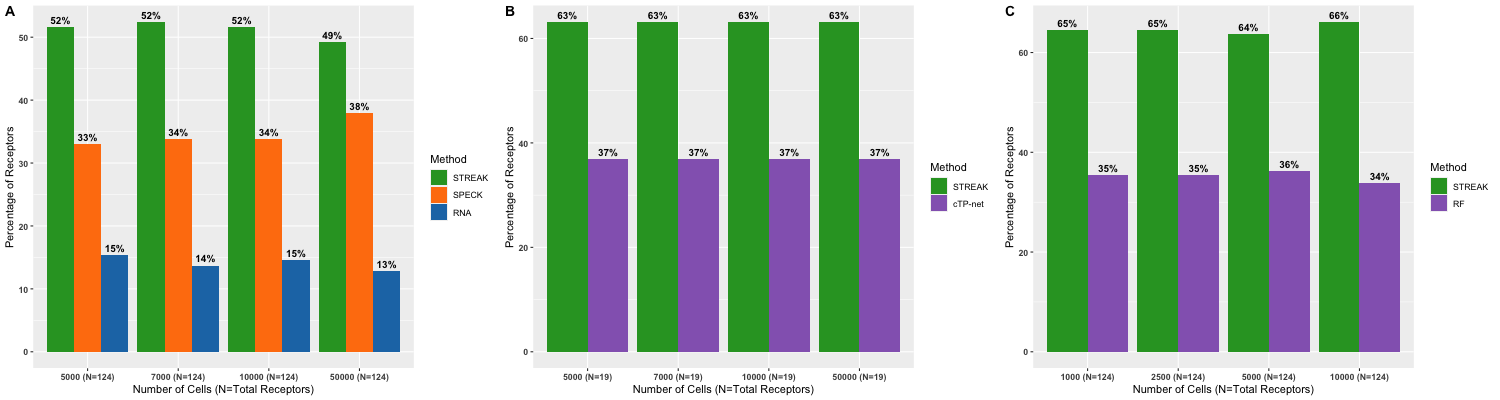

Supplement: S6 Fig — Percentage of receptors with highest average Spearman rank correlations between CITE-seq data and abundance profiles estimated using STREAK, SPECK and normalized RNA approach or cTP-net or RF for the 5-fold cross-validation approach with 5,000 cells from the Hao data for the cross-training evaluation approach (S6A, S6B, S6C). The horizontal axis for these plots (S6A-S6C) indicates the number of target cells evaluated from the Unterman data. (TIFF) [file pcbi.1011413.s006.tiff]

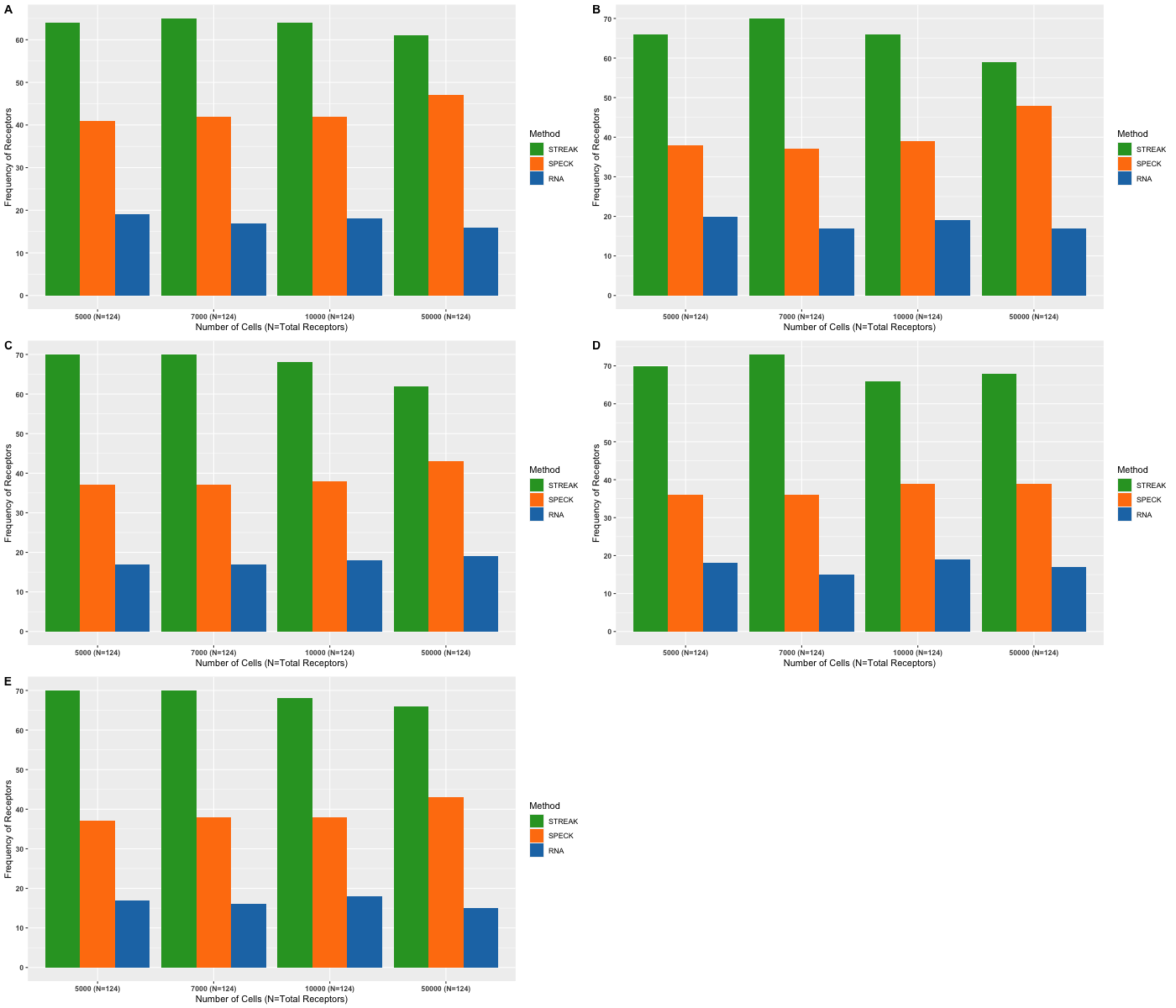

Supplement: S7 Fig — Training data sensitivity analysis examining frequency of receptors with highest average rank correlations between CITE-seq data and abundance values estimated using STREAK, SPECK and normalized RNA transcript evaluated using the cross-training approach with Hao training data consisting of 5,000 (S7A), 7,000 (S7B), 10,000 (S7C), 20,000 (S7D) and 30,000 (S7E) cells. The horizontal axis for each plot indicates the number of target cells evaluated from the Unterman data. (TIFF) [file pcbi.1011413.s007.tiff]

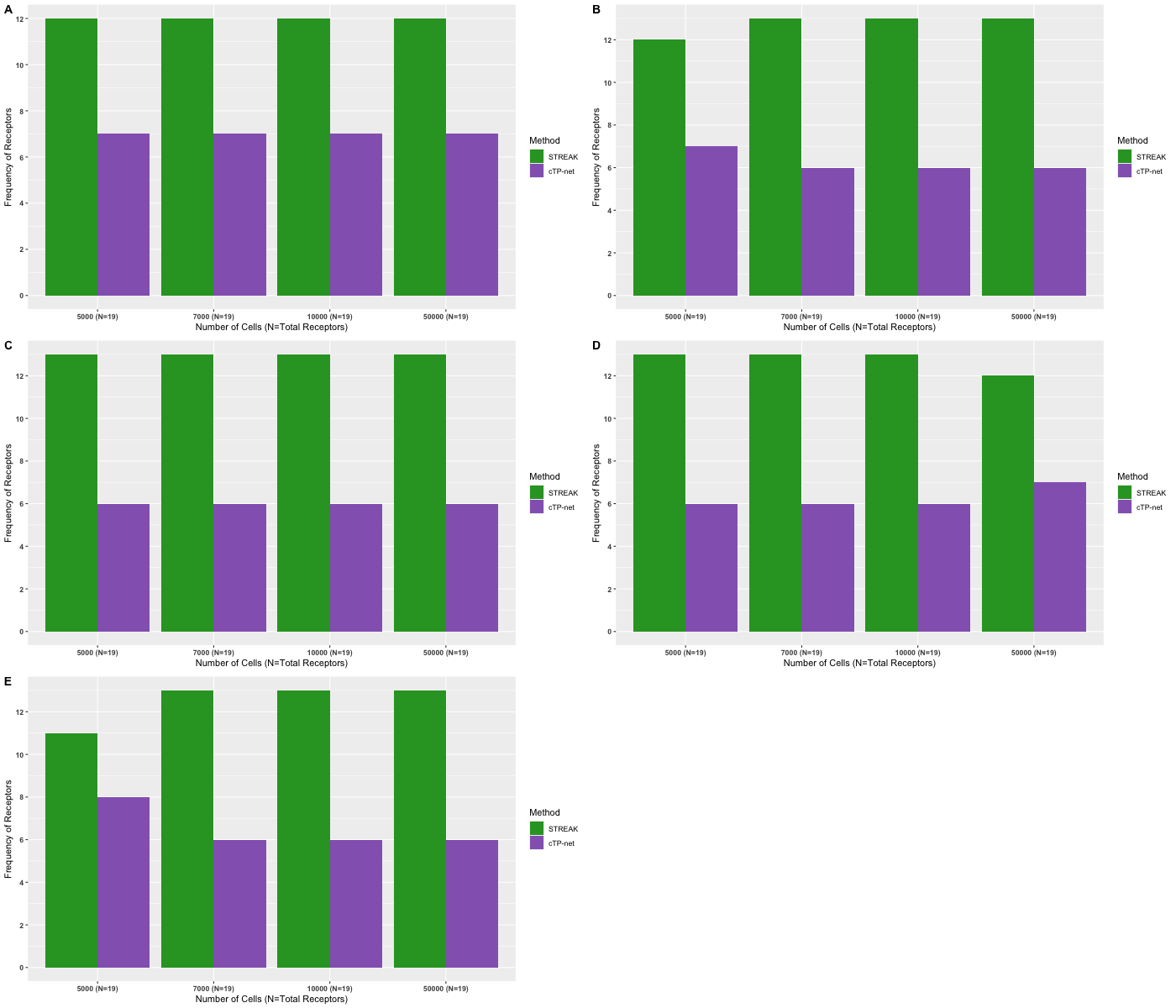

Supplement: S8 Fig — Training data sensitivity analysis examining frequency of receptors with highest average rank correlations between CITE-seq data and abundance values estimated using STREAK and cTP-net via the cross-training strategy. (TIFF) [file pcbi.1011413.s008.tiff]

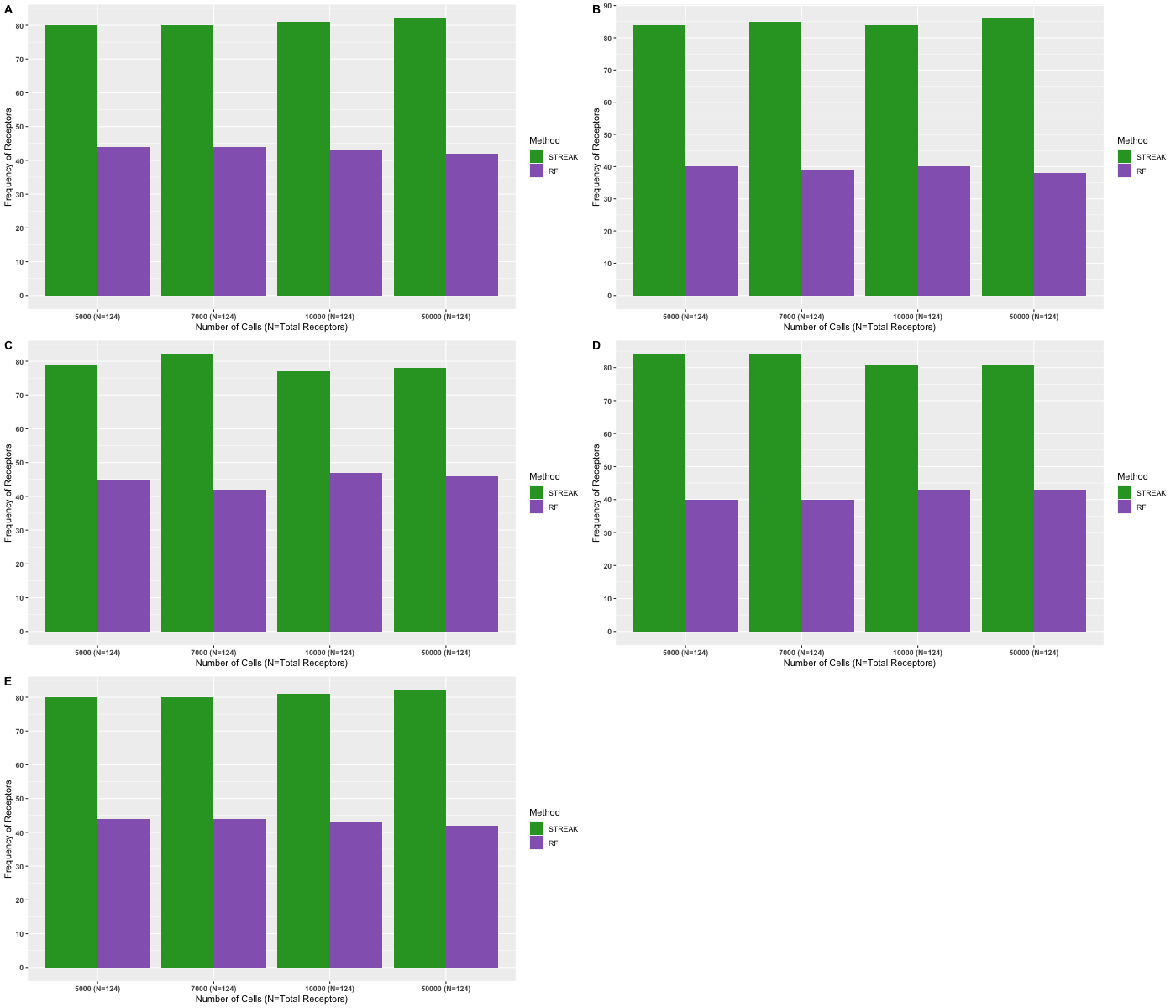

Supplement: S9 Fig — Training data sensitivity analysis examining frequency of receptors with highest average rank correlations between CITE-seq data and abundance values estimated using STREAK and the RF model via the cross-training strategy. (TIFF) [file pcbi.1011413.s009.tiff]

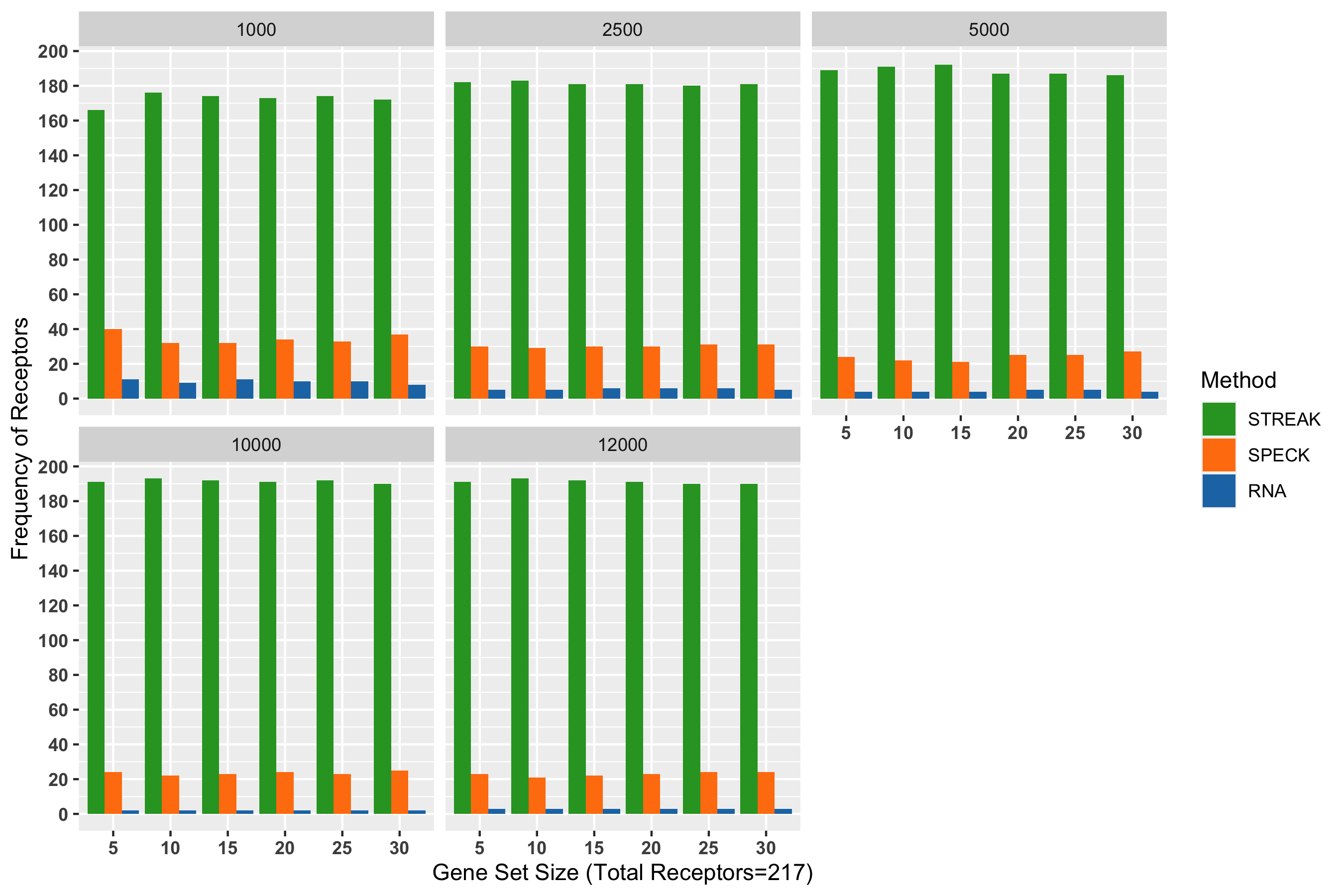

Supplement: S10 Fig — Gene set size sensitivity analysis examining frequency of receptors with highest average rank correlations between CITE-seq data and abundance values estimated using STREAK, SPECK and normalized RNA transcript evaluated using the 5-fold cross-validation approach with the indicated training data ranging from 1,000 to 12,000 cells for the Hao data and gene set size consisting of 5, 10, 15, 20, 25 and 30 genes. (TIFF) [file pcbi.1011413.s010.tiff]

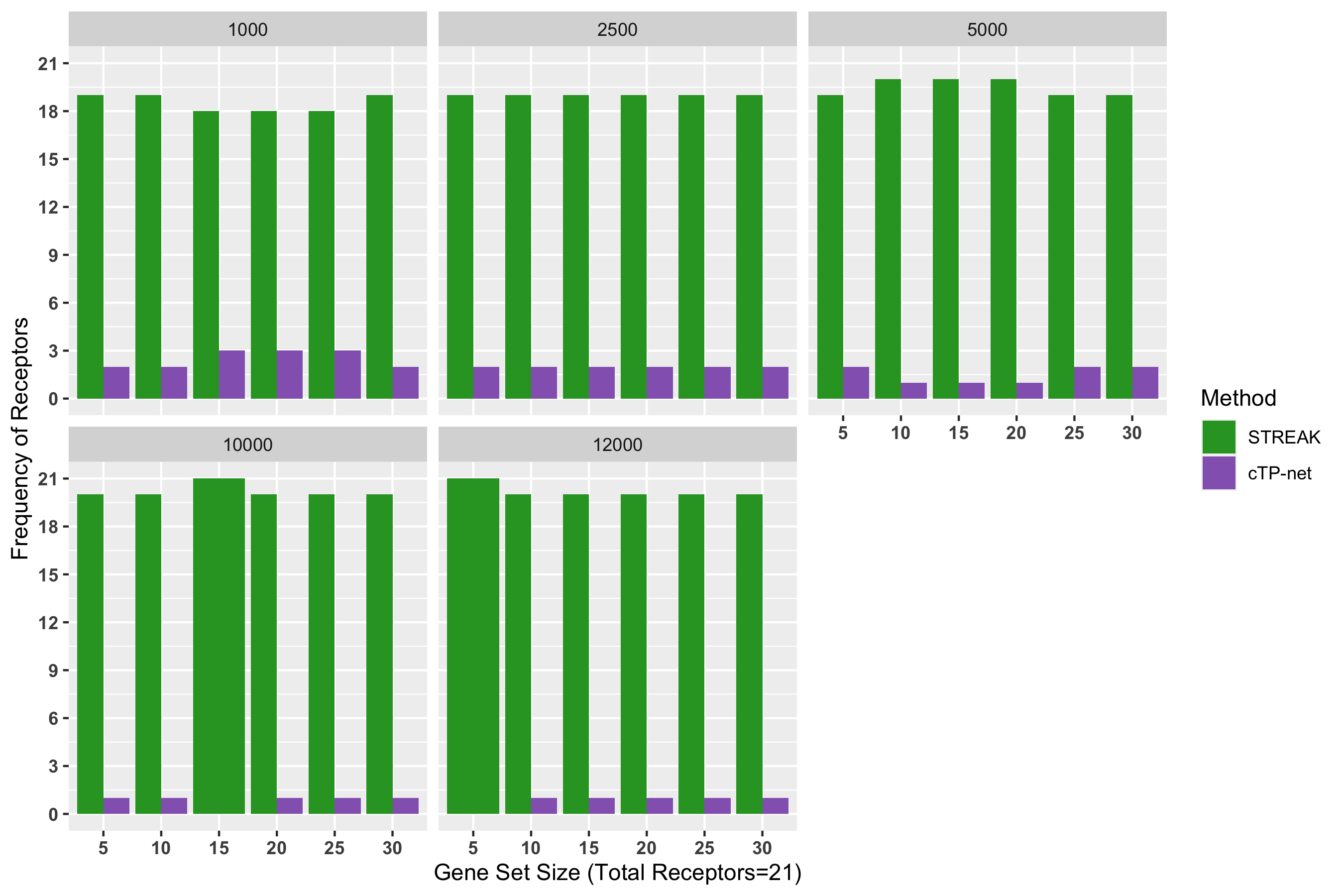

Supplement: S11 Fig — Gene set size sensitivity analysis between CITE-seq data and abundance profiles estimated with STREAK and cTP-net using the 5-fold cross-validation approach with training data ranging from 1,000 to 12,000 cells for the Hao data and gene set size consisting of 5, 10, 15, 20, 25 and 30 genes. (TIFF) [file pcbi.1011413.s011.tiff]

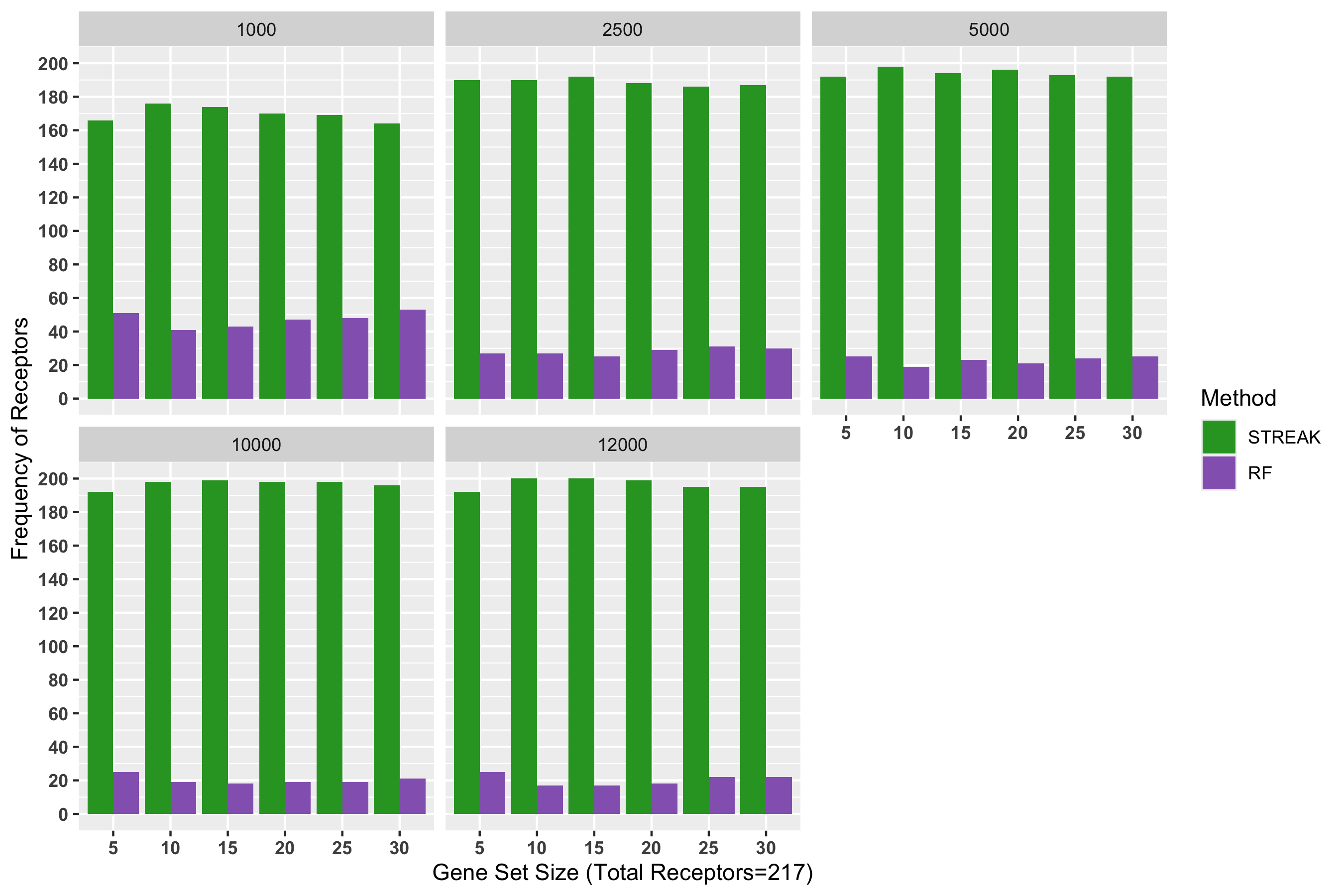

Supplement: S12 Fig — Gene set size sensitivity analysis between CITE-seq data and abundance profiles estimated with STREAK and the RF model using the 5-fold cross-validation approach with training data ranging from 1,000 to 12,000 cells for the Hao data and gene set size consisting of 5, 10, 15, 20, 25 and 30 genes. (TIFF) [file pcbi.1011413.s012.tiff]

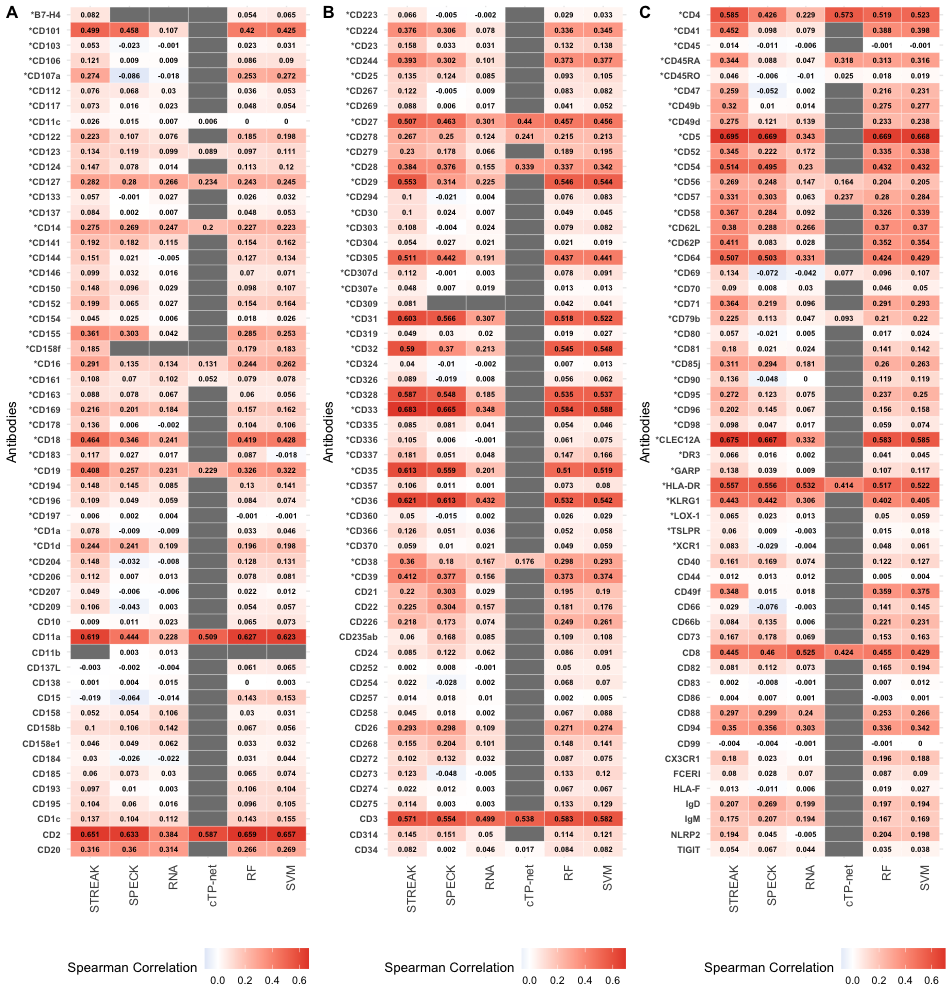

Supplement: S13 Fig — Average rank correlations between CITE-seq data and receptor abundance values estimated using STREAK and comparative methods as evaluated using the 5-fold cross-validation approach for training data consisting of 12,000 cells from the Unterman dataset. (TIFF) [file pcbi.1011413.s013.tiff]

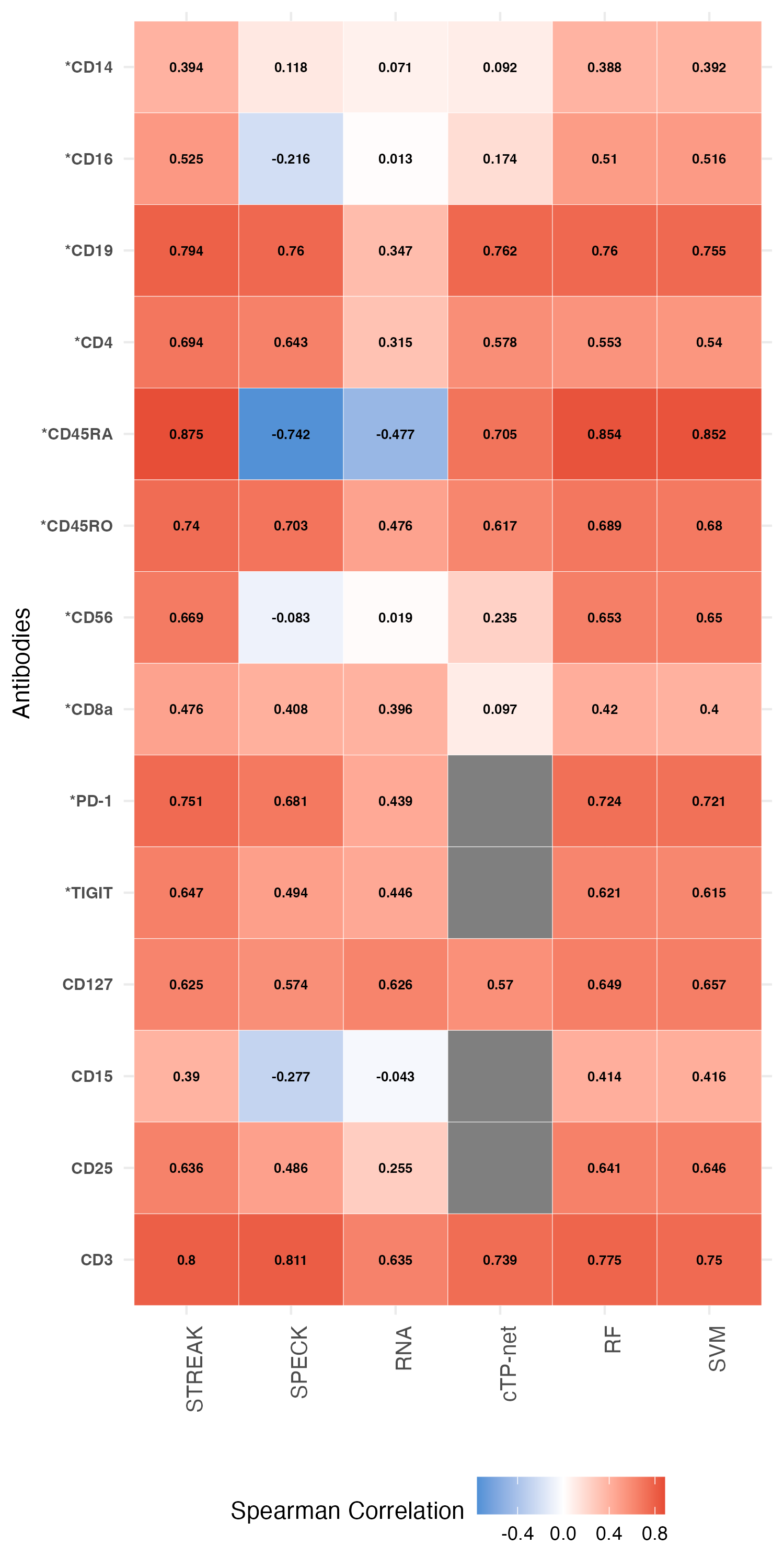

Supplement: S14 Fig — Average rank correlations between CITE-seq data and receptor abundance values estimated using STREAK and comparative methods as evaluated using the 5-fold cross-validation approach for training data consisting of 1,682 cells from the MALT dataset. (TIFF) [file pcbi.1011413.s014.tiff]

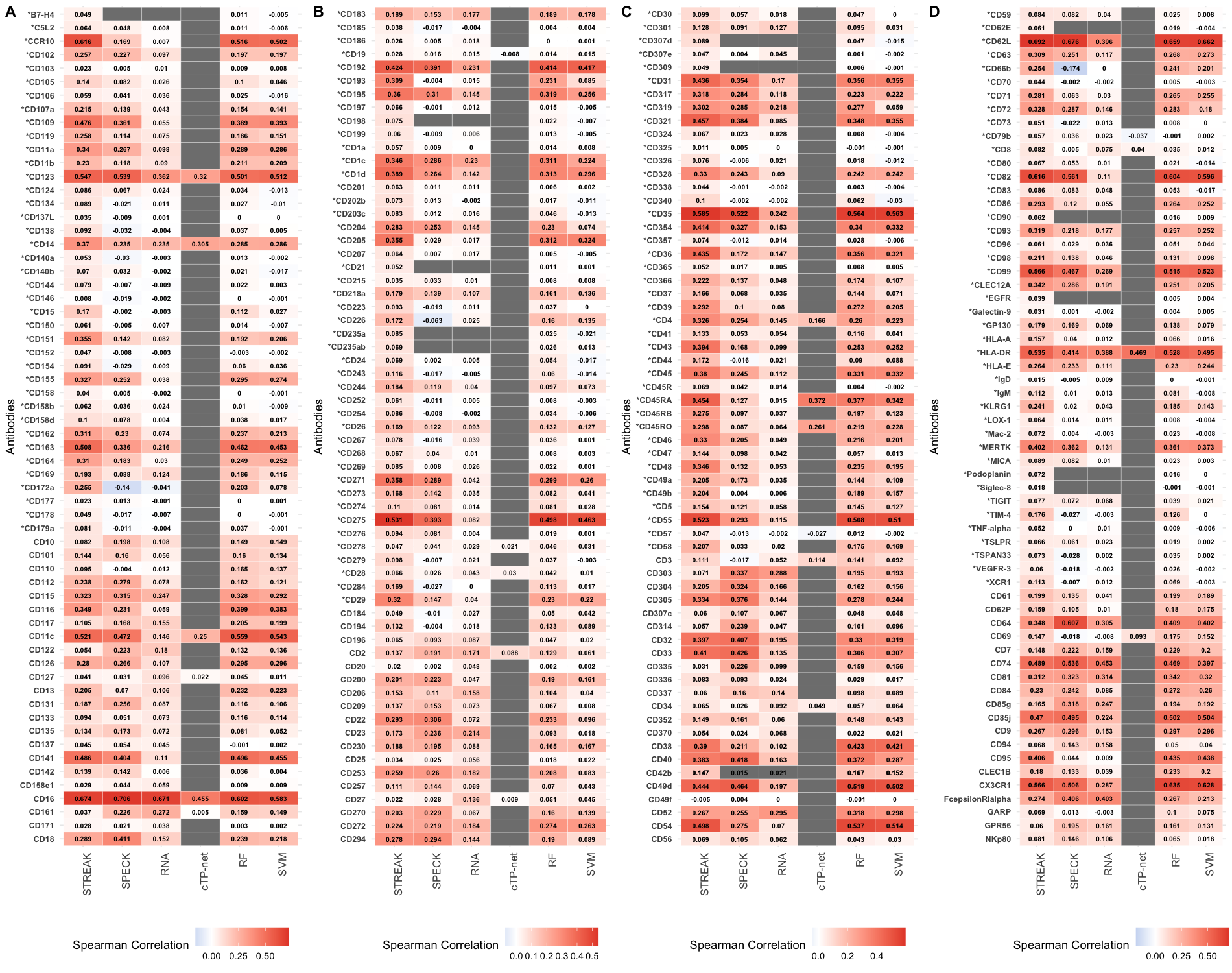

Supplement: S15 Fig — Average rank correlations between CITE-seq data and receptor abundance values estimated using STREAK and comparative methods as evaluated using the 5-fold cross-validation approach for training data consisting of 7,422 cells from the Monocytes dataset. (TIFF) [file pcbi.1011413.s015.tiff]

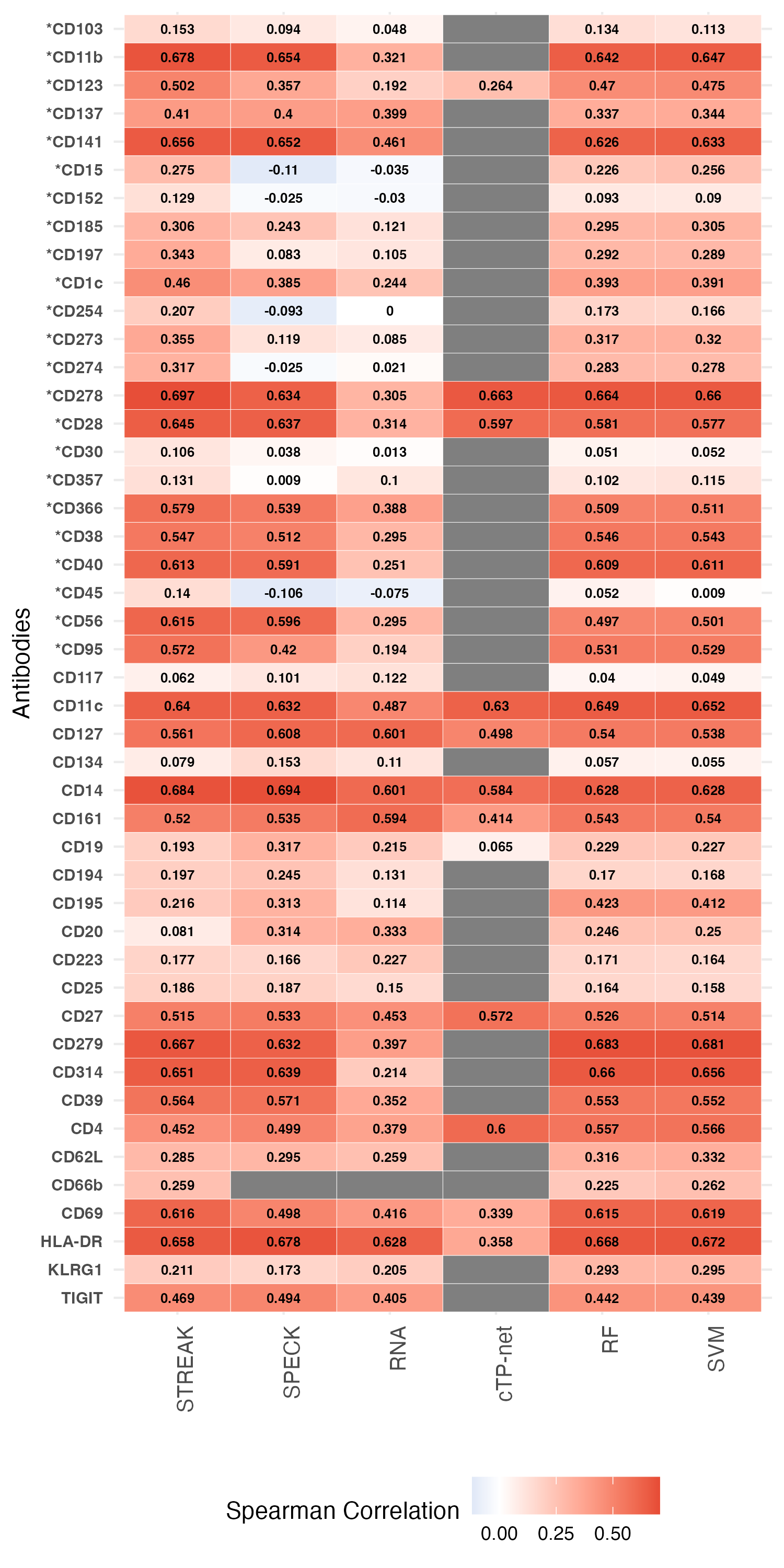

Supplement: S16 Fig — Average rank correlations between CITE-seq data and receptor abundance values estimated using STREAK and comparative methods as evaluated using the 5-fold cross-validation approach for training data consisting of 994 cells from the MPEM dataset. (TIFF) [file pcbi.1011413.s016.tiff]
