# Supplementary material for: STREAK: A supervised cell surface receptor abundance estimation strategy for single cell RNA-sequencing data using feature selection and thresholded gene set scoring
Source: PLoS Comput Biol. 2023 Aug 21;19(8):e1011413. doi: 10.1371/journal.pcbi.1011413 (PMC10470905; doi:10.1371/journal.pcbi.1011413)
Supplement: Attachment — Submitted filename: STREAK-CommentIntegration-AJ-HRF.pdf [file pcbi.1011413.s017.pdf]

Reviewer's Responses to Questions

Comments to the Authors:
Please note here if the review is uploaded as an attachment.

Reviewer #1: In this work, the authors present a supervised method to estimate receptor abundance on individual cells from scRNA-seq data, which is trained on joint scRNA/seq/CITE-seq data. The authors benchmark their method against other state-of-the-art methods designed to address the same problem and show that theirs works better.
The problem the authors are trying to solve is relevant given the recent interest in single-cell multiomics technologies, and the method is well explained and makes sense. However, I found the presentation of the actual results confusing:
1. • For example, several plots rely on the "proportion of abundance profiles that have the highest Spearman rank correlation" metric, which is a hard description to parse. Turns out that this is the number of receptors with the highest correlation with CITE-seq data *compared to the other estimation methods*, which explains trends that are otherwise puzzling like some of the bars going down with increasing numbers of cells in Fig 2. Also the main text uses the word "proportion" but the plots use "frequency", and actually show absolute numbers in their y axes. If this metric is to be used, it should be explained better and with more consistent terminology. Furthermore, figs. 3-6 show that in several cases, even though STREAK has higher performance, the difference is not that large. This makes it questionable to use a metric based on which method has the highest correlation. In other words, there is a loss of information of the actual correlation values in figs. 2, 7, 8, and 9 that I think is important.

<u>Response:</u> Thank you for raising this issue. We have adjusted the bar charts (as displayed by Figures 2-6) to more consistently display the percentage of receptors that are estimated with STREAK and are most correlated with analogous CITE-seq data among the comparative approaches.

3. The inclusion of the different numbers of cells in Figs 2, 7, and 9 is interesting in principle, but no insight is extracted or discussed in the text. Fig. 8 is the exception, where the different numbers of cells used for training have a clear purpose. But even then, the point of using different number of cells for their test dataset is unclear.

<u>Response:</u> Thank you for your comment. We agree with your interpretation. We have updated the manuscript to present a final Figure for just the total number of cells, as indicated by Figures 2 and 3. We originally included comparisons for different sized random subsets of cells to examine how the method scales to variations in cell subset size. These subset-based results have been moved into the supplemental material, as indicated by Supplementary Figure 5.

4. Figs 3-6 contain tables with every estimation for every receptor in every dataset, which makes it hard to parse or extract any insights from them.

Response: Thank you for your comment. We have adjusted these tables per your suggestion. We originally included these tables since we wanted to present the raw correlations between the estimated abundance profiles and the corresponding CITE-seq data. We have removed some of the heatmaps to the supplement section.

5. Finally, the plot text (axis and tick labels) is too small, which makes it impossible to read on printed paper and requires lots of zooming in a computer.
I would recommend the authors to use correlation vs correlation scatter plots, with each point corresponding to a receptor and the x and y axis showing the spearman correlations between CITE-seq and each one of the estimation methods under study. The position of the dots can be compared with the x=y diagonal to see which method is overall better for most receptors, and numerical information about the correlation values is not lost. This makes for a more succinct comparison of correlation performance metrics, and maybe the number of figures and panels can be reduced. For examples, check Fig. 1b of https://www.nature.com/articles/s41592-021-01252-x.

Response: We appreciate the Reviewer's excellent suggestion. We have implemented the correlation scatterplots in our updated version of the manuscript as indicated by Figures 7 and 8 for the PBMC Hao and Mouse Spleen datasets, respectively, and Supplementary Figures 1, 2, 3 and 4 for the PBMC Unterman, MALT, Monocytes and the MPEM datasets, respectively.

Finally, while the selection of competing methods against which this work is compared is adequate, the authors avoid comparing against PIKE-R2P because, they claim, the code was not available. As of this writing the code seems to be available at https://github.com/JieZheng-ShanghaiTech/PIKE-R2P. Therefore, I would ask the authors to perform this comparison.

Response: Thank you for your comment. We made additional attempts to include PIKE-R2P in our evaluation framework but were unfortunately not successful. The PIKE-R2P method is not currently appropriately packaged via either the Python or R code repository platforms. Moreover, all code dependencies are not adequately documented, and the documented code is not currently set up to run with python3.

For example, when we run the .sh file after modifying the python version to python3, we observe that downstream packages such as sklearn need to be individually loaded for our machine. Once that package is loaded, we get a subsequent error for a different missing package. While we could set up an anaconda environment to specifically customize our environment, we would need the exact Python package and subsequent dependencies information appropriately detailed. By comparison, we were able to package the downstream dependencies for our method into a single R package (now

available via both the CRAN and Github computing frameworks) such that they can be easily loaded and executed on different computing devices.

We believe that the current set of five comparison methods (RNA transcript, SPECK, SVM, Random Forests, and cTP-net) are a good representation of both unsupervised and supervised approaches. However, if the reviewers feel that additional comparison methods are needed, we are open to adding results for methods such as the ADImpute, which is a recently published, and ease to use, semi-supervised imputation framework.

Reviewer #2: I read this paper entitled “STREAK: A Supervised Cell Surface Receptor Abundance Estimation Strategy for Single Cell RNA-Sequencing Data using Feature Selection and Thresholded Gene Set Scoring” with great interest, as it proposes a new solution to the challenges associated with estimating receptor abundance for scRNA-seq target data.
The authors have developed a new supervised receptor abundance estimation method called STREAK for single-cell transcriptomics data. This method is an improvement over their previous unsupervised method SPECK, as it leverages associations learned from joint scRNA-seq/CITE-seq training data and uses a thresholded gene set scoring mechanism. The authors evaluate STREAK against both unsupervised and supervised methods on three joint scRNA-seq/CITE-seq datasets and conclude that it outperforms other techniques and provides a more biologically interpretable and transparent statistical model.
I commend the authors for the introduction of a new and improved method for cell surface receptor abundance estimation. The use of joint scRNA-seq/CITE-seq training data to leverage associations and improve the accuracy of the estimation is one of the strengths. Besides, the authors have also carried out a detailed evaluation of the method on multiple datasets and a demonstration of its superiority over other techniques.

I have following questions/suggestions for the authors:

1. How logical and fair is the comparison with other techniques given those methods may have different assumptions and limitations, particularly the comparison between supervised and unsupervised techniques?

Response: Thank you for your very well-reasoned question. Reviewer 2 is correct to note that both supervised and unsupervised techniques have a different set of assumptions. Please see below regarding our assessment on the limitations and assumptions of comparative techniques that motivated STREAK. First, while the simple approach of replacing gene expression values with the RNA transcript generally functions well, it is a low-quality approximation due to the associated sparsity issues of the mRNA transcript. As an alternative to this approach, while a cluster-based approach that functions by averaging transcript expression over large populations/clusters of cells generally functions well, it has two major limitations. First, it assumes that receptor

abundance is uniform across all cells in each cluster. Second, this approach ignores cluster-level heterogeneity.

This latter limitation motivated the development of alternative, unsupervised receptor abundance estimation approaches such as the SPECK method. SPECK makes a low-dimensionality assumption that leverages gene expression redundancy to perform abundance estimation/imputation. A key limitation of SPECK is that it does not generalize well to all receptors. SPECK additionally assumes that transcriptomics is a proxy for proteomics, which is biologically sound (as reported in our SPECK paper available on the bioRxiv server).

While the limitations above can be remedied by leveraging supervised receptor abundance estimation approaches such as cTP-net, there are several associated limitations with this approach. First, the cTP-net approach is limited in that it only currently functions for 24 immunophenotype markers/receptors. Second, and, more importantly, cTP-net utilizes a deep learning model that is trained on the same training population as the test population. Therefore, it does not correctly capture out-of-sample error/variance and related expression patterns that generalize to non-immune-related surface markers. Lastly, like other deep learning approaches, the cTP-net approach is limited in that it is not very customizable, and, therefore, not very interpretable.

Given the assumptions and limitations detailed above, we developed STREAK and the associated STREAK R package. In future, we plan to leverage STREAK's capabilities to perform an assessment on individual receptors and extend its applicability to different classes of cell-surface receptors, such as the G protein-coupled receptors. This will allow us to then test for cell-cell communication, functional crosstalk between receptors and perform investigation in the resulting, downstream intracellular signaling propagation.

In terms of the fairness of comparing supervised and unsupervised approaches, we agree that supervised approaches should in general perform better than unsupervised approaches. We included both types of techniques in our evaluation of STREAK to demonstrate that a supervised method does indeed outperform unsupervised methods on relevant datasets. This type of evaluation is especially important in cross-training scenarios where supervised methods have more risk of overfitting the training data.

2. The evaluation of the method is limited to three joint scRNA-seq/CITE-seq datasets representing two different tissue types and using two evaluation strategies. In my opinion, a wider range of data and a larger number of datasets should be used to validate the generalizability and robustness of the method. Can we use or expand the same method to other tissue types and bigger datasets?

Response: Thank you for your comment. We have expanded STREAK's applicability to the Monocytes, Mouse Spleen and Lymph Nodes, and the Malignant Peritoneal Mesothelioma (MPEM) datasets. Thus, in total, we have expanded STREAK's

applicability to six datasets, which include the PBMC Hao, PBMC Unterman, MALT, Monocytes, Mouse Spleen and Lymph Nodes, and the MPEM datasets.

3. I would appreciate it if the authors can prepare and highlight the comparison of different methods in a Tabular format as a Main Table.

Response: Thank you for your comment. We have included two Tables currently. Table 1 details a list of all six joint scRNA-seq/CITE-seq datasets used for method evaluation while Table 2 details the number of cells used in individual training and target data subsets as well as the total number of cells for each of the six datasets. Please let us know if this is sufficient.

Reviewer #3: in the manuscript "STREAK: A Supervised Cell Surface Receptor Abundance Estimation Strategy for Single Cell RNA-Sequencing Data using Feature Selection and Thresholded Gene Set Scoring: the authors report on a new supervised receptor abundance estimation method.

The manuscript and the tool are of definite interest and I have only minor comments:

1. Would be possible to apply this tool to generate accurate estimation of cell surface receptor abundance for tissue-specific single cell data beyond blood / immune cells?

Response: Thank you for this suggestion. We have extended STREAK's implementation to additional datasets of different tissue types. Currently, we have expanded STREAK's applicability to the Monocytes, Mouse Spleen and Lymph Nodes, and the Malignant Peritoneal Mesothelioma (MPEM) datasets. Thus, in total, we have expanded STREAK's applicability to six datasets, which include the PBMC Hao, PBMC Unterman, MALT, Monocytes, Mouse Spleen and Lymph Nodes, and the MPEM datasets.

2. The authors evaluated STREAK against the Random Forest (RF) algorithm, but RF heavily depends on feature selection. Can the authors evaluate the data with at least one more machine learning algorithm such as SVM or GBM to confirm STREAK superiority to other machine learning methods.

Response: We thank the Reviewer for this suggestion. We have extended STREAK's evaluation against the SVM method. We report that STREAK performs better than R's SVM implementation from the e1071 package. Please see Figures 2, 3, 5 and 6 for the data regarding STREAK's comparison against the SVM technique. Additionally, we extend STREAK's implementation on three additional datasets, which include the Monocytes, MPEM and the Spleen and Lymph Nodes datasets.

3. The authors say that they did not compare STREAK against PIKE-R2P as it is not in CRAN, but the R code is available in github. Comparison should be conducted.

Response: As noted above, the PIKE-R2P method is not currently appropriately packaged via either the Python or R code repository platforms. Moreover, all code dependencies are not adequately documented, and the documented code is not currently set up to run with python3.

Additionally, when we run the .sh file after modifying the python version to python3, we observe that downstream packages such as sklearn need to be individually loaded for our machine. Once that package is loaded, we get a subsequent error for a different missing package. While we could set up an anaconda environment to specifically customize our environment, we would need the exact Python package and subsequent dependencies information appropriately detailed. By comparison, we were able to package the downstream dependencies for our method in a single R package (now available via both the CRAN and Github computing frameworks) such that they can be easily loaded and executed on different computing devices.

We believe that the current set of five comparison methods (RNA transcript, SPECK, SVM, Random Forests, and cTP-net) are a good representation of both unsupervised and supervised approaches. However, if the reviewers feel that additional comparison methods are needed, we are open to adding results for methods such as the ADImpute, which is a recently published, semi-supervised imputation framework.

4. The CDF in the manuscript had gamma distribution, but single cell data usually follows a negative binomial distribution. Is there an advantage to use gamma distribution?

Response: Thank you for your comment. The VAM method, which is the gene set scoring technique integrated into the STREAK algorithm, uses a gamma distribution to fit the squared modified Mahalanobis distances that are computed on sets of normalized scRNA-seq values. A gamma distribution is fit to the squared distances for a permuted version of the data and this null distribution is then used to compute CDF values for non-permuted squared distances. While the reviewer is correct in noting that unnormalized scRNA-seq counts generally follow a negative binomial distribution, squared Mahalanobis distances computed on sets of normalized counts do not follow this distribution. Per the central limit theorem, these squared distances will be approximately chi-square distributed (they have an exact chi-square distribution if the data is normal). As detailed in the VAM paper (https://academic.oup.com/nar/article/48/16/e94/5868339), we found that the more flexible gamma distribution provides a better fit for these squared distances when sparsity levels are high, which is typically the case for scRNA-seq data.
