# Supplementary material for: STREAK: A supervised cell surface receptor abundance estimation strategy for single cell RNA-sequencing data using feature selection and thresholded gene set scoring
Source: PLoS Comput Biol. 2023 Aug 21;19(8):e1011413. doi: 10.1371/journal.pcbi.1011413 (PMC10470905; doi:10.1371/journal.pcbi.1011413)
Supplement: Attachment — Submitted filename: STREAK-CommentIntegration-AJ-HRF-June26.pdf [file pcbi.1011413.s018.pdf]

Reviewer #1: Thanks to the authors for addressing my comments. I still have one important concern and a few presentation issues:

1. • The authors mention that they were unable to run PIKE-R2P from its github repo. However, the main text still says “We do not perform comparison against PIKE-R2P since its implementation does not currently exist on the Comprehensive R Archive Network (CRAN) or on alternative code repository platforms”, which is untrue. The authors should update this statement and describe, briefly but precisely, what issues they had with the software.

Response: Thank you for your comment. We have modified our original statement appropriately as “We do not perform comparison against PIKE-R2P since, while an initial implementation exists on Github, the upstream package dependencies are not adequately documented.”

2. • From the description of their algorithm in the methods section, is seems that the VAM method only uses X*_R but not A. This is important since the authors claim that one advantage of their method is that the gene weights can be interpreted, or manually tuned to reflect biological knowledge. Can the authors clarify where A is used?

Response: Thank you for your comment and we have updated the text associated with Step 2 of the VAM method overview in Section 3.3.1 to clarify how matrix A is leveraged. To summarize, Matrix A is the gene set membership matrix with each column of A corresponding to a gene set (one per receptor), each row corresponding to a gene, and element i,j set to 0 if gene i is not a member of set j or a positive weight if gene i is a member of set j. The STREAK method leverages the information in matrix A to 1) determine the set of genes used to compute the modified Mahalanobis distances for each receptor and 2) to adjust the technical variance of each gene used in the distance calculation. Specifically, the technical variance is divided by the weight to yield larger Mahalanobis distances for genes with larger weights.).

3. • Minor clarification: In step 2 of their summary of VAM, M[,k] is supposed to be a column matrix with dimensions (m2, 1) but the matrix product is Xk^T*(Ig*sigma^2)^-1*X_k, which is (m2, g)^T*(g, g)* (m2, g) = (g, m2)*(g, g)*(m2, g), which doesn’t make sense for a matrix product. Is this wrong, or is there a typo?

Response: Thank you for pointing this out. This was a typo and has been appropriately corrected. In this case, M[,k] is set to the diagonal elements of the following matrix product: $M[,k] = diag(X_{k}(I_{g}\sigma^2_{g,tech})^{-1}X_{k}^{T})$.

4. • While I appreciate that the bar plots have been simplified, I’m concerned that some of them do not show all the bars that they should. For example the MALT dataset in Figure 2 does not include bars for the RNA and RF methods. From looking at the supplementary figures, it seems that their value is zero, but this is not obvious from the figures. Maybe the authors can modify the plots to make this more explicit, or at the very least include a note in the figure legend saying that all bars unshown correspond to a value of zero.

Response: We have updated all barplots to account for zero percentage.

5. • The authors say that heatmaps use bold text to indicate receptors where STREAK estimates are higher than other methods. But this is 1) really hard to see, 2) from what I can tell there are several receptors in which STREAK performs best that are not bolded. Example: CD11b-2 in Figure 9a. Can you clarify?

Response: Thank you for pointing this out. In our previous evaluation strategy, we compared STREAK's performance across all receptors for which cTP-net was defined, which resulted in a small number of receptors for which STREAK performed best given the relatively small sample size. We have now modified our approach such that we compare against cTPnet if the results are present and compare STREAK between the remaining strategies when cTPnet is not defined. We have additionally updated the heatmaps to use asterisks to indicate receptors where STREAK performs best according to the evaluation criteria delineated above.

6. • Minor: In Figure 13, the y axis is frequency instead of proportion.

Response: Thank you, we have appropriately adjusted the Figure.

Reviewer #2: I have carefully reviewed the revised manuscript titled "STREAK: A Supervised Cell Surface Receptor Abundance Estimation Strategy for Single Cell RNA-Sequencing Data using Feature Selection and Thresholded Gene Set Scoring" along with responses to my previous comments, and I recommend accepting it for publication in PLOS Computational Biology.

I appreciate the thoroughness with which the authors addressed my concerns and incorporated the suggested revisions. The revised manuscript now provides a clear and comprehensive description of the STREAK method for estimating cell surface receptor abundance in single-cell RNA-sequencing data.

Reviewer #3: No further comments
